# Cost-consequence analysis of the enhanced recovery after surgery protocol in major lung resection with minimally invasive technique (VATS)

**DOI:** 10.3389/fsurg.2024.1471070

**Published:** 2024-10-30

**Authors:** Alessandra Buja, Giuseppe De Luca, Stefano Dal Moro, Marco Mammana, Anna Zanovello, Stefano Miola, Deris Gianni Boemo, Ilaria Storti, Pietro Bovo, Fabio Zorzetto, Marco Schiavon, Federico Rea

**Affiliations:** ^1^ Department of Cardiological, Thoracic, Vascular Sciences and Public Health, University of Padua, Padua, Italy; ^2^ Department of Directional Hospital Management, Padua University Hospital, Padova, Italy; ^3^ Management Control Unit, Padua University Hospital, Padova, Italy

**Keywords:** lung cancer, cost analysis, cost consequence analysis, health care services, health economics, ERAS, VATS

## Abstract

**Background:**

ERAS is an evidence-based multimodal perioperative protocol focused on stress reduction and promoting a return to function. The aim of this work is to perform a cost-consequence analysis for the implementation of ERAS in major lung resection by means of minimally invasive surgery (VATS) from the public health service perspective, evaluating resource consumption and clinical outcomes with respect to a control group of past patients, which did not adopt an ERAS protocol.

**Methods:**

Outcome differences (re-intervention rates, major and minor intraoperative and postoperative complications, readmissions, and mortality) as well as the costs of preoperative, operative, and postoperative care were estimated. The sample consisted of 64 consecutive patients enrolled in the ERAS programme between April 2021 and August 2022, compared to a control group (historical cohort) comprising 31 patients treated from April 2020 to December 2020, prior to the implementation of the ERAS programme. The study sample comprises patients who fulfil the established ERAS protocol inclusion criteria, including general criteria (acceptance of the protocol, proximity of residence, absence of contraindications to physiotherapy and early mobilisation), surgical criteria (anatomical lung resection up to lobectomy, absence of extensive resection, good possibility of conducting the operation in VATS) and anaesthesiologic criteria (ASA ≤2). Costs were quantified using the national health system perspective.

**Results:**

The average length-of-stay was at least one day shorter in the ERAS group [<0.001. Average total costs including entire pathway healthcare costs were substantially reduced for ERAS-VATS patients (mean: € 5,955.71 vs. €6,529.41 Δ = −573.70 *p* = 0.018)]. Specifically, the median costs of the admission phase were significantly different between the two groups (median: €4,648.82 vs. €5,596.58, *p* = 0.008), with a reduction in hospital stay expenditure in the ERAS-VATS group (median: €1,599.62 vs. €2,399.43, *p* = 0.025). No significant differences were found regarding major clinical outcomes.

**Conclusions:**

The implementation of an ERAS programme is a dominant strategy, representing an intervention capable of reducing overall costs in the context of elective anatomical lung resection with VATS without any significant differences in major complications and re-intervention rates.

## Introduction

Despite the advances observed in thoracic surgery, lung anatomical resections (lobectomy or segmentectomy) remain an invasive and traumatic procedure, especially in elderly and frail lung cancer patients. In this context, minimally invasive Video-Assisted Thoracoscopic Surgery (VATS) has become the procedure of choice when treating early-stage lung cancer (1, 2). Nonetheless, this type of intervention still causes surgical stress and has significant side effects, and is thus associated with considerable postoperative issues, the most common of which is cardio-pulmonary morbidity (3). ERAS is an evidence-based multimodal perioperative protocol focused on stress reduction and the promotion of a return to function. By combatting harmful stress response, ERAS reduces one of the main pathogenic factors leading to morbidity in surgical patients (4) When examining the mechanisms underlying the efficacy of ERAS, it can be seen that homeostasis is often maintained by controlling metabolism and fluids, thus supporting the recovery of key functions (5, 6). Other fundamental elements of ERAS approach include, among the others, preoperative counselling, smoking and alcohol cessation, carbohydrate loading, avoidance of preoperative sedatives, prevention of hypothermia, regional anaesthesia, postoperative nausea and vomiting (PONV) control, opioid-sparing analgesia, early chest drain removal, and early mobilization after surgery (7).

This enhanced recovery pathway applies to the patient's whole journey from referral to discharge, via the coordinated actions of a multidisciplinary team working together to ensure the synergic application of all elements of the programme at every stage of care (pre-, intra- and post-operative) (8).

Several meta-analyses have investigated the outcomes obtained when using ERAS protocols in different surgery fields. In thoracic surgery, a systematic review and metanalysis of ERAS has recently demonstrated a strong benefit of ERAS implementation on postoperative hospital LOS, diminished by 3 days, and modest benefit on readmission rates (9). Although ERAS programmes have been shown to improve patient outcomes in several types of surgery also reducing costs, few studies have investigated the cost-effectiveness of an ERAS protocol in VATS surgery (10–14), and even fewer from the perspective of public national healthcare services. Moreover, little is known on the impact of ERAS protocol implementation on VATS surgery after the discharge.

From an economic point of view, it was reported that lung cancer surgical treatment has a similar cost in both the early and the advanced stages (15). An ERAS program to help reduce side effects associated with lung surgery would thus result in shorter hospital stays and lower treatment costs.

The aim of this work is to perform a cost-benefit analysis for the implementation of ERAS in major lung resection by means of minimally invasive surgery (VATS), with a dedicated patient telemonitoring platform for the follow-up of patients after discharge, evaluating resource consumption and clinical outcomes compared to a control group of past patients (who did not adopt an ERAS protocol).

## Materials and methods

### Setting

The present study was carried out in the Thoracic Surgery Operative Unit of Padua University Hospital (Azienda Ospedale—Università Padova, AOUP, Italy), a high-volume university hospital with more than 200 minimally invasive anatomic lung resections performed every year.

### Study design

A cost-consequence analysis was conducted to estimate the outcomes (re-intervention rates, major and minor intraoperative and postoperative complications, readmissions, and mortality) and the costs of patients undergoing major lung resection by means of minimally invasive surgery (VATS) with an ERAS protocol (including both a completed-ERAS group, and a discontinued-ERAS group), compared to patients that didn't have an ERAS protocol. Sensitivity analysis was also conducted considering just the completed-ERAS group, compared to the control group. The time horizon of the study includes the preoperative period (from the preoperative surgical visit), the operative period and the postoperative care period of the patients (up to 40 days after discharge, coinciding with the time of the post-discharge visit).

### The ERAS protocol

ERAS plays a role over the patient's entire course of treatment, which is divided into three phases: pre-operative, peri-operative, and post-operative (as summarized in Supplementary Table 1). The 45 recommendations of the Enhanced Recovery After Surgery (ERAS) Society and the European Society of Thoracic Surgeons (ESTS) were taken into account when defining the ERAS protocol applied by the Thoracic Surgery Unit (7). In addition to these, our ERAS protocol includes a further distinctive element: postoperative telemonitoring. This was offered to patients who were discharged within the first three post-operative days, with the aim of providing protracted contact with treating physicians and additional patient comfort. The telemonitoring platform was set up specifically for this ERAS program, with access given only to ERAS patients and continuing for 15 days after discharge. The main features of the platform included daily collection of physiologic parameters using standardized forms (e.g., body temperature, heart rate, oxygen saturation, pain score), shared educational content (dealing with the overall purpose of ERAS and videos of physiotherapy exercises), as well as facilitated contact with the Thoracic Surgery Unit through an instant messaging feature.

### Sample

The study group (ERAS) consisted of 64 consecutive patients enrolled in the ERAS programme for major lung resection up to lobectomy (excluding bilobectomy) between April 2021 and August 2022, all of whom met the criteria defined for inclusion in the ERAS protocol (Table 1).

**Table 1:** ERAS protocol inclusion criteria.

General criteria
• The patient is deemed capable of understanding the protocol and respecting it in all its parts, especially where active participation is required • The patient, after being adequately informed, accepts all the components of the protocol, including early discharge and eventual discharge with chest drainage • Geographical proximity • No contraindications to physiotherapy and early mobilisation^a^
Surgical criteria
• Anatomical lung resection up to lobectomy (bilobectomy excluded) • Very good chance of conducting the operation using a minimally invasive technique (VATS) • Any established or presumed diagnosis (both malignant and benign disease) • No extensive resections (diaphragm, ribs)
Anaesthesiological criteria
• American Society of Anesthesiologists (ASA) ≤2

^a^ Concomitant medical conditions that limit, contraindicate or prevent peri-operative mobilisation (e.g., paraplegia or impairment of one or more limbs.). In doubtful cases, assessment by the physiatrist is required.

**Figure 1:**
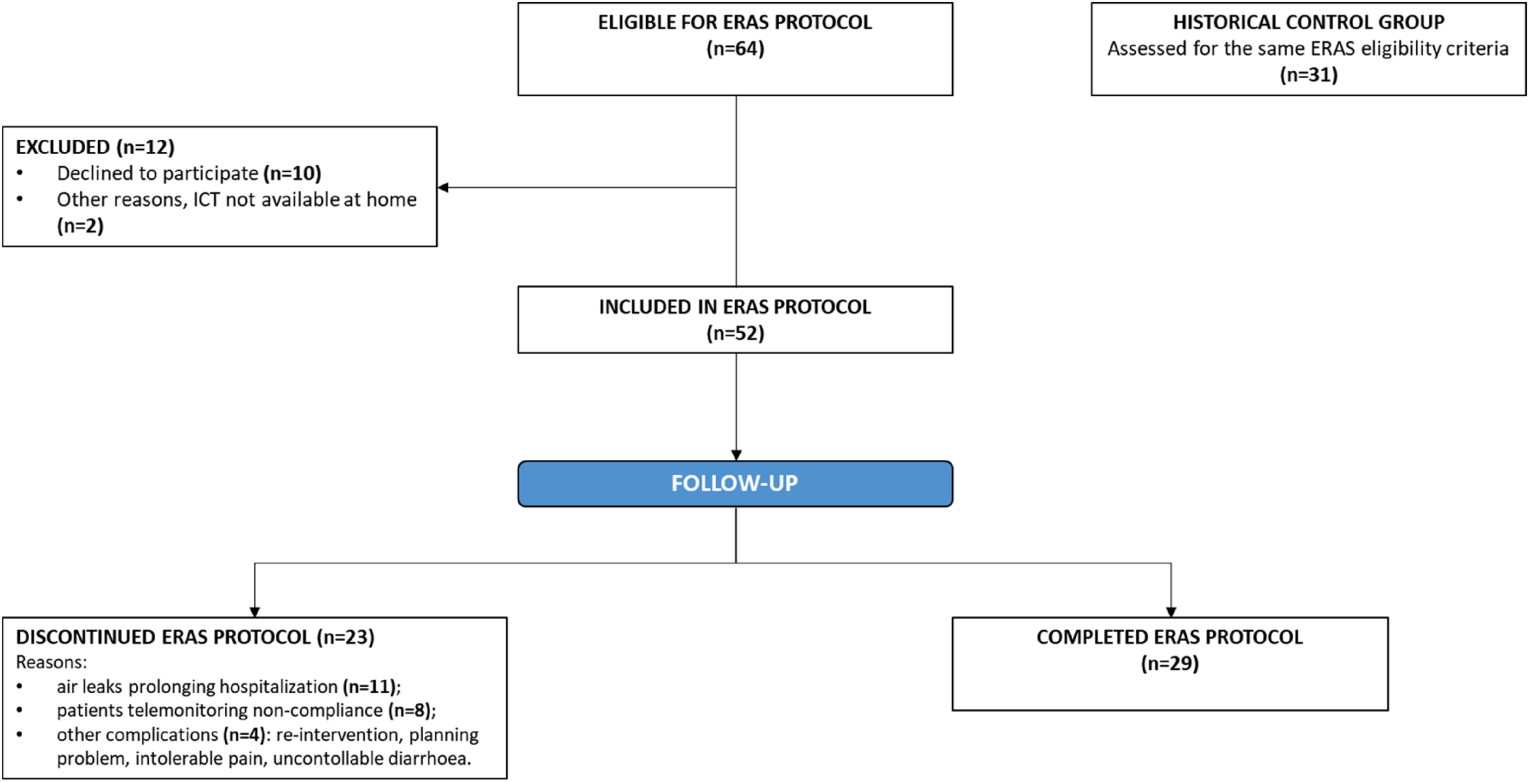
Intervention groups and control group.

Among these patients, 12 refused to participate (motivations: technological obstacles, declined consent) (Figure 1). Among the 52 ERAS patients, 23 discontinued the protocol. Reasons for protocol non-compliance included persistent air leaks prolonging hospitalization (11 patients), telemonitoring non-compliance (8 patients), other complications (a re-intervention, a planning problem, intolerable pain, and uncontrollable diarrhoea; 4 patients). The percentage of non-compliance to ERAS protocol is coherent with several lung surgery survey, which have shown different grades of compliance with ERAS principles (16, 17).

The control group consisted of a consecutive set of 31 past patients identified through the hospital's electronic databases from April 2020 to December 2020 (i.e., prior to the implementation of the ERAS programme), all of whom met the same inclusion criteria used for the ERAS group. Patients in the control group, despite having surgery without ERAS, had some important features in common with patients in the ERAS group, including minimally invasive surgical procedures, a single chest drain, and early chest drain removal. No changes were made to the surgical procedures or in during this period. They also received standard preoperative-operative and postoperative care.

The main similarities and differences in peri-operative management between patients in the ERAS group and control group as summarized in Supplementary Table 2.

## Materials

Since the implementation of ERAS, data have been collected in a dedicated database, while control group data were collected retrospectively.

The data collected included patients’ demographic characteristics, principal diagnosis, comorbidity index (measured with Charlson's Comorbidity Index (CCI) (18), Barthel Index (BI) (19), pre-operative and 15-day post-operative Barthel Dispnea Scor (20), quality of life measurement (using Short Form Health Survey SF-36 Questionnaire (21), and 1-month postoperative complications follow-up. Postoperative complications were classified according to the Clavien-Dindo classification and adapted to thoracic surgery, considering grade I–II as minor complications and grade III–IV as major complications, with grade V considered as 30-day postoperative mortality (22).

All laboratory analyses, imaging, other diagnostic procedures, medications, or blood transfusions administered to patients during preoperative care (starting with the preoperative visit that assessed the patients’ suitability for surgery) and postoperative care (ending with the 40-day follow-up visit to monitor patients after surgery) were collected by reviewing medical records, including a number of specialist medical and physiotherapy visits. The length of stay and details of the surgery, such as the type of anatomical lung resection and the duration of the operation, were also recorded.

### Cost analyses

The costs were evaluated in the perspective of regional health service, in particular the costs were based on the reimbursement rates established by the Veneto Regional Authority. The health resources included for cost estimation were laboratory and other diagnostic tests, medical examinations, and medications and other consumables. To convert resource use into monetary terms, we multiplied the resources consumed by their unit cost, using the tariffs reported in the Nomenclatore Tariffario delle Prestazioni Ambulatoriali (NTPA), a regional outpatient tariff list. Retail prices were used to calculate the financial value of the medications and consumables.

The cost of staying one day in the surgical ward were estimated by the hospital administration. It was obtained by dividing total hospitalization costs for the year in that ward by the total number of inpatient days produced by this ward. The general and indirect costs associated with hospital functioning, such as depreciation, repairs, maintenance, expansions, food, laundry, utilities, and insurance, were identified and included in full admission costs.

The fixed cost regarding the digital platform for postoperative telemonitoring of the discharged patients account for a total amount of € 15,000: cost of the platform (13,750 EUR) and the costs of the digital video for patient education (750 EUR of doctor's time and 500 EUR of nurse's time) and the time for educating the ward's doctors in the use of the platform (100 EUR for 10 doctors). Due to the time taken by the doctors, which was only a few seconds per day to view the alerts highlighted in the platform we, we did not take this time into account and also three 10-min phone calls with patients to check for altered or missing parameters (variable costs).

### Statistical analysis

Absolute frequencies and percentages were used for the descriptive statistics of categorical variables, while continuous numerical variables were represented by means, median, standard deviations (SD), and interquartile range (IQR). The Chi-square test was applied to identify differences in the distribution of categorical variables. Fisher's test was only used when there was at least one cell with a value less than five in the contingency table. *T*-test or Kolmogorov-Smirnov test was used to compare continuous variables, as appropriate (normal distribution was assessed using the Shapiro-Wilk normality test). A *p*-value of <0.05 was considered statistically significant. All statistical analyses were performed using the R 3.6.2 statistical package.

### Ethical statement

The study was conducted in accordance with the principles established in the Declaration of Helsinki. The study protocol was approved by the local ethics board and individual consent was waived (No. Prot. Rev.0 from 05 to 08-22).

## Results

The main clinical and pathological characteristics of the ERAS and non-ERAS groups are summarized in Table 2, showing no difference in sociodemographic or clinical variables distribution, no difference in comorbidity index, no difference in characteristics tumours by group, except for the ERAS group having a more advanced-stage lung cancer distribution.

**Table 2:** Characteristics of patients undergoing VATS anatomical lung resections by ERAS groups.

	Controls	ERAS	*p*-value
	(*n* = 31, 35%)	(*n* = 52, 65%)	
Baseline characteristics			
Age (mean and IQR)	63.74 (58–73),	62.5, (54.5–72),	0.939
Sex (Female), *n* (%)	14, (45.16%)	26, (50.00%)	0.842
Body mass index (BMI) (kg/m2) (mean and IQR)	26.05 (23.2–28.01)	25.80 (22.85–28.27)	0.785
Smoker			
No, *n* (%)	11 (35.48%)	20 (38.46%)	0.082
Yes, *n* (%)	15 (48.39%)	14 (26.92%)	
Former, *n* (%)	5 16.13%)	18 (34.62)	
ASA Group			
1, *n* (%)	0 (0%)	2 (3.85%)	0.526
2, *n* (%)	31 (100%)	50 (96.15%)	
Barthel score (mean and IQR)	95.32 (90–100)	98.46 (100–100)	0.215
Charlson comorbidity index (mean and IQR)	3.71 (2–5)	3.81 (3–5.25)	0.745
Malignancy, *n* (%)	29 (93.55%)	49 (94.23%)	1
Histotype			
NSCLC adenocarcinoma, *n* (%)	25 (80%)	33 (63.46%)	0.368
NSCLC squamous cell carcinoma, *n* (%)	1 (3.23%)	5 (9.62%)	
NSCLC adenosquamous	1 (3.23%)	2 (3.85%)	
Carcinoid	4 (12.90%)	6 (11.54%)	
Metastasis	0 (0%)	1 (1.92%)	
Other, *n* (%)	0 (0%)	5 (9.62%)	
Stage (of malignancies)			
I	28 (90.32%)	28 (53.85%)	0.015
II	2 (6.45%)	11 (21.15%)	
III	1 (3.23%)	8 (15.38%)	
IV	0 (0%)	1 (1.92%)	
Missing	0 (0%)	4 (7.69%)	
Neoadjuvant therapy, *n* (%)			
No, *n* (%)	30 (96.77%)	51 (98.08%)	1
QoL questionnaire (pre-admission)			
Physical functioning (median and IQR)	85 (61–91)	85 (65–95)	0.997
Pain (median and IQR)	61 (55–92)	70 (55–90)	0.973
Emotional wellbeing (median and IQR)	57 (48–65)	64 (60–84)	0.517
Social functioning (median and IQR)	62 (50–75)	75 (50–75)	0.892
General health (median and IQR)	45 (40–51)	55 (50–70)	0.021
Surgical intervention variables			
Resection, *n* (%)			
Segmental resection	19 (61.29%)	20 (38.46%)	0.074
Lobectomy	12 (38.71%)	32 (61.54%)	
Operating time (minutes) (mean and IQR)	120.00 (100–130)	103.20 (75–120)	0.013
Length of Stay (days) (mean and IQR)	7.13 (5–8)	5.67 (3–6)	<0.001

The average length of stay was at least one day shorter in the ERAS group **(**Table 2**)**.

Table 3 shows the costs per phase of the surgical pathway for both the ERAS and non-ERAS groups. The median pre-admission cost wasn't significantly different (median: €460.50 vs. €369.55, *p* = 0.068) for the ERAS-VATS group and the control group. The median costs of the admission phase were significantly different between the two groups (median: €4,648.82 for ERAS-VATS vs. €5,596.58 for the control, *p* = 0.008), with a reduction in hospital stay costs in the ERAS-VATS group (median: €1,599.62 vs. €2,399.43, *p* = 0.025). In contrast, no differences were seen in the post-admission phase. Average total costs including entire pathway healthcare costs were substantially reduced for ERAS-VATS patients (mean: € 5,955.71 vs. €6,529.41 Δ = −573.70 *p* = 0.018). It would then be necessary to deal with nearly 26 patients to cover the fixed costs arising from the digital platform.

**Table 3:** Cost of the patient undergoing VATS anatomical lung resections before and after ERAS implementation.

	Controls		ERAS (*n* = 52, 65%)		Δ Median	*p* value
	(*n* = 31, 35%)					
	Median	STD. dev	Median	STD. dev		
Pre-admission	369.55	264.06	460.50	520.04	90.95	0.068
Medications	0.00	0.00	0.00	111.47	0	0.764
Examination, visits, imaging	369.55	264.06	460.50	483.69	90.95	0.119
Full admission including allocated costs	5,596.58	1,439.03	4,648.82	1,233.83	−947.76	0.008
Hospital stay	2,399.43	1,262.71	1,599.62	1,142.99	−799.81	0.025
Examination, visits, imaging	524.01	207.21	518.26	181.65	−5.75	0.409
Medications	122.58	109.50	69.57	66.12	−53.01	<0.001
Medical devices	9.00	3.71	29.65	2.83	20.65	<0.001
Post-admission	299.62	539.93	167.22	224.07	−132.4	0.127
Medications	65.87	27.97	60.74	21.41	−5.13	0.011
Examination, visits, imaging, telemonitoring	233.75	540.55	122.65	225.58	−111.1	0.186
Total costs	6,280.85	1,606.99	5,631.84	1,358.08	−649.01	0.018

The results of sensitivity analysis supported the conclusion that the costs of all surgical pathway phases were different between the completed-follow-up ERAS group and the non-ERAS group. Data confirmed that the median of the pre-admission phase costs was higher for ERAS patients (median: €460.50 Eras vs. €369.55 non-ERAS, *p* = 0.014), with a decrease in the admission phase (median: €4,514.61 vs. €5,596.58, *p* < 0.001) and post-admission phase (median: €143.67 vs. €299.62, *p* = 0.304) costs for completed-ERAS subjects. Overall, the resulting total costs were lower for the ERAS group (mean: €5,554.33 vs. €6,529.41, Δ = −975.08, *p* = 0.005). It would then be necessary to deal with nearly 15 patients to cover the fixed costs arising from the digital platform.

The main clinical outcomes analysed are shown in Table 4. No significant differences were found between the two groups compared.

**Table 4:** Outcomes of patient undergoing VATS anatomical lung resections before and after ERAS implementation.

	Control group	ERAS group	*P* value
	(*n* = 31)	(*n* = 52)	
30-day mortality	0 (0%)	0 (0%)	–
Intra-operative complications (*n*.%)	0 (0%)	1 (1.92%)	1
Post-operative complications during hospitalisation (*n*.%)	4 (12.90%)	13 (25.00%)	0.263
Minor (Clavien-Dindo I–II) (*n*.%)	2 (6.45%)	10 (19.23%)	0.195
Major (Clavien-Dindo III–IV) (*n*.%)	2 (6.45%)	3 (5.77%)	1
Surgical re-intervention (*n*.%)	1 (3.23%)	1 (1.92%)	1
Complications after discharge (*n*.%)	1 (3.23%)	4 (7.69%)	0.646
Hospital re-admission	0 (0%)	0 (0%)	–
Combined outcomes	6 (19.35%)	18 (34.62%)	0.211

When considering just the completed-ERAS group, the number of patients with complications during the pathway was 6 (20.69%) and no significant difference was observed when compared to the control group (*p* = 1).

## Discussion

The present work compared patient care with elective anatomical lung resection through Video Assisted Thoracic Surgery (VATS) before and after the implementation of the ERAS program. We found that overall costs after the implementation of ERAS were lower, together with a reduction in length of stay (LOS). These data are consistent with a recent metanalysis which reported that the implementation of an ERAS program for surgery of lung cancer can effectively reduce risks of postoperative complications, length of stay, and costs of patients who have undergone lung cancer surgery without compromising their safety (23).

In the pre-admission phase, the intervention group (ERAS-VATS) had a slight and not significant increase in costs compared to the controls. These higher costs can be attributable to medications in this early phase of treatment as the ERAS program includes malnutrition correction through the prescription of an immune-nutritional drink for 7 days before the surgery, in order to reduce complications and accelerate post-operative recovery, as recommended by international clinical guidelines (24–27).

The admission phase shows a decrease of almost €947 (17%) in the ERAS group median cost. The observed reduction in LOS in the ERAS group strongly contributed to lowering the costs associated with hospitalization (reduction in median cost €-799.81). The evidence of a shorter hospitalization time is line with the significant reduction in post-operative LOS after the implementation of ERAS protocols in VATS (4.0 days vs. 6.0 days and 6.58 vs. 8.69) reported by Martin et al. in the US and Huang et al. in China (12, 28). Similar results were found the meta-analysis indicated that patients in the ERAS group had a significantly shortened postoperative length of stay (SMD = −1.58; 95% CI: −2.38 to −0.79) and in also with the results in other surgical fields, such as elective colorectal surgery, as reported in the systematic review by Greer et al. (29).

In the post-admission phase, no differences in costs were shown between the intervention and control groups. This is consistent with the absence of significant differences in major complications and hospital readmission rates, similar to what has been reported in previous studies for ERAS implementation in VATS surgery (12, 28) and in a recent metanalysis showing that significant reduction was found in the readmission rate (RR = 1.00; 95% CI: 0.76–1.32).

Overall, the analysis indicates that ERAS program implementation is an influential strategy, saving around €650 (10%) in median treatment costs with no differences in relevant clinical outcomes. This is consistent with the data available on ERAS implementation in VATS as reported in recent studies from Switzerland and the US, which showed a significant reduction in mean total costs of €4.766 (11) and €6.067 (12), respectively. This cost-saving effect of ERAS is also similar to what has been observed in other surgical fields after the introduction of enhanced recovery protocols, including gynaecological (30, 31), colorectal (32, 33), gastric (34, 35), pancreatic (36, 37) and oesophageal surgery (38, 39).

## Limitations

The limitations of this study relate to the fact that it focuses only on direct costs, as it is based on real hospital data and adopts a health system perspective, and consequently does not include indirect costs (i.e., related to lost productivity, including the opportunity cost of informal caregivers’ time) and direct non-medical costs (e.g., transport). Other limitations are the sample size considered and the retrospective nature of the analysis, although a comprehensive prospective of patient baseline characteristics is presented to exclude selection bias.

## Conclusions

Our study has demonstrated that the implementation of an ERAS programme is capable of reducing overall costs in the context of elective anatomical lung resection with a VATS approach in a university hospital setting. The reduction of the length of stay is the main factor in reducing overall costs. Additionally, no significant differences in major complications and re-intervention rates were found.

## Data Availability

The raw data supporting the conclusions of this article will be made available by the authors, without undue reservation.
