# Supplementary material for: Cost-consequence analysis of the enhanced recovery after surgery protocol in major lung resection with minimally invasive technique (VATS)
Source: Front Surg. 2024 Oct 30;11:1471070. doi: 10.3389/fsurg.2024.1471070 (PMC11557562; doi:10.3389/fsurg.2024.1471070)
Supplement: Supplementary file 1 [file Table1.docx]

**Supplementary Table 1.** Major peri-operative, intra-operative, and post-operative areas of intervention in the ERAS protocol.

| Pre-operative | Intra-operative | Post-operative |
| --- | --- | --- |
| Patient education | Warming of the patient | Early mobilization |
| Avoidance of prolonged fasting | ESP block. avoidance of epidural | Early feeding |
| Carbohydrate loading | “Balanced” fluid regimen | Avoidance of urinary catheter |
| Immunonutrient supplementation | Minimally invasive approach | Standardized chest tube removal criteria |
|  | Single chest drain | Telemonitoring |

| **Item** | **ERAS Protocol** | **Control group** |
| --- | --- | --- |
| **Pre-operative period** | | |
| Nutritional evaluation and immunonutrition | Yes | No |
| Screening from anemia and iron supplementation | Yes | No |
| Education to physiotherapy exercises | Yes | No |
| Preoperative fasting | 6 hours before surgery for solids  2 hours before surgery for fluids | Fasting starts midnight the day before surgery |
| Carbohydrate loading | Yes | No |
| **Intraoperative period** | | |
| Urinary catheter | No | Yes |
| Opioid-sparing analgesia | Yes | No |
| Regional anesthesia type | ESP block | Epidural analgesia or ESP |
| Minimally invasive surgery | Yes | Yes |
| Single chest drain | Yes | Yes |
| **Postoperative period** | | |
| Fluid therapy | 1000 ml the day of surgery | 2000 ml daily from POD0 to 2 |
| Mobilization | Starting the day of surgery | Usually on POD1 |
| Physiotherapy | Starting the day of surgery | Usually on POD2 |
| Opioid-sparing analgesia | Yes | No |
| Resumption of oral nutrition | 2 hours after surgery for fluids  6 hours after surgery for solids | On POD1 for fluids  On POD2 for solids |
| Antibiotic prophylaxis | Single dose 2 hours before surgery | During hospitalization and 6 days after discharge |
| Early drain removal | Yes | Yes |
| Tele-monitoring | Yes | No |

**Supplementary Table 2.** Main similarities and differences between the ERAS protocol and the control group

ERAS: enhanced recovery after surgery; ESP: erector spinae plane; POD: postoperative day
